# Supplementary material for: Adaptively Integrative Association between Multivariate Phenotypes and Transcriptomic Data for Complex Diseases
Source: Genes (Basel). 2023 Mar 26;14(4):798. doi: 10.3390/genes14040798 (PMC10138055; doi:10.3390/genes14040798)

Web-based Supplementary Materials for "Adaptively integrative association between multivariate phenotypes and transcriptomic data for complex diseases" by Yujia Li, Yusi Fang, Hung-Ching Chang, Michael Gorczyca, Peng Liu and George C. Tseng

March 26, 2023

Table S1: Results of Simulation IA and IB when $N_1 = 30$. For $\sigma_\mu = 0$, the value indicates Type I error control, and for $\sigma_\mu = 0.4$ and 0.6, the value indicates power.

Benchmark	Method	Simulation IA			Simulation IB		
		σ_μ=0	σ_μ=0.4	σ_μ=0.6	σ_μ=0	σ_μ=0.4	σ_μ=0.6
power & type I error	MANOVA	0.05	0.1	0.23	0.05	0.4	0.68
	aSPU.ind	0.04	0.14	0.33	0.04	0.16	0.36
	aSPU.ex	0.04	0.13	0.31	0.04	0.16	0.35
	TATES	0.05	0.08	0.21	0.05	0.39	0.6
	minP	0.05	0.1	0.27	0.05	0.44	0.65
	Fisher	0.05	0.14	0.37	0.05	0.4	0.57
	AFz	0.05	0.12	0.33	0.05	0.44	0.65
	AFp	0.05	0.13	0.35	0.05	0.43	0.64
Sensitivity	AFz	-	0.17	0.31	-	0.26	0.31
	AFp	-	0.18	0.33	-	0.3	0.43
Specificity	AFz	-	0.81	0.79	-	0.83	0.85
	AFp	-	0.81	0.78	-	0.8	0.77

Table S2: The average weight estimate for AFp and AFz methods for Simulation I. For example, for the AFp method, when $\sigma_\mu = 0.6$ and $N_1 = 100$, the average weight estimate for genes 1–1600 and 2th phenotype $\boldsymbol{Y_2}$ is 0.72 among 500 simulated data. Since the weight can be either 0 or 1, it means 72% of the weights for $\boldsymbol{Y_2}$ and genes 1–1600 are estimated to be 1 among 500 simulated datasets.

N	σ_μ	Method	Gene sets	$\boldsymbol{Y_1}$	$\boldsymbol{Y_2}$	$\boldsymbol{Y_3}$	$\boldsymbol{Y_4}$	$\boldsymbol{Y_5}$	$\boldsymbol{Y_6}$	$\boldsymbol{Y_7}$	$\boldsymbol{Y_8}$	$\boldsymbol{Y_9}$	$\boldsymbol{Y_{10}}$
$N_1 = 30$	$\sigma_\mu = 0.4$	AFp	$\boldsymbol{X_1} - \boldsymbol{X_{1600}}$	0.56	0.15	0.16	0.15	0.28	0.14	0.15	0.16	0.15	0.09
			$\boldsymbol{X_{1601}} - \boldsymbol{X_{3200}}$	0.07	0.1	0.1	0.09	0.48	0.14	0.14	0.14	0.16	0.1
			$\boldsymbol{X_{3201}} - \boldsymbol{X_{4800}}$	0.09	0.1	0.1	0.1	0.09	0.09	0.09	0.1	0.09	0.32
		AFz	$\boldsymbol{X_1} - \boldsymbol{X_{1600}}$	0.55	0.1	0.11	0.1	0.19	0.09	0.1	0.11	0.11	0.06
			$\boldsymbol{X_{1601}} - \boldsymbol{X_{3200}}$	0.06	0.08	0.09	0.08	0.47	0.12	0.12	0.12	0.13	0.08
			$\boldsymbol{X_{3201}} - \boldsymbol{X_{4800}}$	0.08	0.09	0.09	0.09	0.08	0.08	0.09	0.09	0.08	0.3
	$\sigma_\mu = 0.6$	AFp	$\boldsymbol{X_1} - \boldsymbol{X_{1600}}$	0.64	0.29	0.32	0.3	0.48	0.26	0.28	0.3	0.28	0.09
			$\boldsymbol{X_{1601}} - \boldsymbol{X_{3200}}$	0.07	0.09	0.1	0.08	0.61	0.23	0.23	0.24	0.25	0.1
			$\boldsymbol{X_{3201}} - \boldsymbol{X_{4800}}$	0.08	0.09	0.1	0.1	0.08	0.08	0.09	0.09	0.08	0.68
		AFz	$\boldsymbol{X_1} - \boldsymbol{X_{1600}}$	0.62	0.14	0.16	0.15	0.17	0.12	0.14	0.15	0.14	0.04
			$\boldsymbol{X_{1601}} - \boldsymbol{X_{3200}}$	0.06	0.07	0.08	0.06	0.6	0.17	0.18	0.18	0.2	0.07
			$\boldsymbol{X_{3201}} - \boldsymbol{X_{4800}}$	0.07	0.08	0.09	0.08	0.07	0.07	0.07	0.08	0.07	0.66
$N_1 = 100$	$\sigma_\mu = 0.4$	AFp	$\boldsymbol{X_1} - \boldsymbol{X_{1600}}$	0.67	0.34	0.34	0.35	0.65	0.33	0.32	0.32	0.32	0.1
			$\boldsymbol{X_{1601}} - \boldsymbol{X_{3200}}$	0.07	0.1	0.09	0.09	0.66	0.31	0.3	0.3	0.31	0.09
			$\boldsymbol{X_{3201}} - \boldsymbol{X_{4800}}$	0.09	0.1	0.1	0.09	0.08	0.09	0.1	0.11	0.1	0.75
		AFz	$\boldsymbol{X_1} - \boldsymbol{X_{1600}}$	0.65	0.15	0.15	0.15	0.19	0.14	0.14	0.14	0.14	0.04
			$\boldsymbol{X_{1601}} - \boldsymbol{X_{3200}}$	0.05	0.05	0.05	0.05	0.64	0.18	0.16	0.17	0.18	0.05
			$\boldsymbol{X_{3201}} - \boldsymbol{X_{4800}}$	0.07	0.08	0.08	0.07	0.07	0.07	0.08	0.09	0.08	0.74
	$\sigma_\mu = 0.6$	AFp	$\boldsymbol{X_1} - \boldsymbol{X_{1600}}$	0.86	0.72	0.71	0.72	0.84	0.68	0.68	0.67	0.67	0.1
			$\boldsymbol{X_{1601}} - \boldsymbol{X_{3200}}$	0.08	0.1	0.1	0.1	0.82	0.67	0.65	0.66	0.67	0.09
			$\boldsymbol{X_{3201}} - \boldsymbol{X_{4800}}$	0.08	0.09	0.1	0.08	0.06	0.08	0.09	0.1	0.09	0.99
		AFz	$\boldsymbol{X_1} - \boldsymbol{X_{1600}}$	0.78	0.28	0.28	0.28	0.25	0.24	0.24	0.25	0.24	0.03
			$\boldsymbol{X_{1601}} - \boldsymbol{X_{3200}}$	0.03	0.03	0.03	0.03	0.76	0.29	0.28	0.29	0.3	0.03
			$\boldsymbol{X_{3201}} - \boldsymbol{X_{4800}}$	0.02	0.03	0.02	0.02	0.02	0.02	0.02	0.03	0.02	0.99

Table S3: Results of Simulations IIA and IIB when $N = 100$. For $\sigma_\mu = 0$, the value indicates Type I error control, and for $\sigma_\mu = 0.4$ and 0.6, the value indicates power.

Benchmark	Method	Simulation IIA			Simulation IIB		
		σ_μ=0	σ_μ=0.4	σ_μ=0.6	σ_μ=0	σ_μ=0.4	σ_μ=0.6
Power & Type I error	MANOVA	0.35	0.53	0.86	0.78	0.78	0.94
	aSPU.ind	0.05	0.42	0.67	0.05	0.45	0.69
	aSPU.ex	0.05	0.41	0.67	0.05	0.44	0.7
	TATES	0.05	0.25	0.78	0.06	0.75	0.96
	minP	0.05	0.33	0.84	0.05	0.77	0.97
	Fisher	0.05	0.43	0.87	0.05	0.71	0.88
	AFz	0.05	0.4	0.9	0.05	0.77	0.97
	AFp	0.05	0.42	0.9	0.05	0.76	0.96
Sensitivity	AFz	-	0.34	0.59	-	0.29	0.31
	AFp	-	0.37	0.71	-	0.48	0.77
Specificity	AFz	-	0.78	0.76	-	0.86	0.89
	AFp	-	0.76	0.68	-	0.73	0.67

Table S4: Results of Simulations IIA and IIB when $N_1 = 30$. For $\sigma_\mu = 0$, the value indicates Type I error control, and for $\sigma_\mu = 0.4$ and 0.6, the value indicates power.

Benchmark	Method	Simulation IIA			Simulation IIB		
		σ_μ=0	σ_μ=0.4	σ_μ=0.6	σ_μ=0	σ_μ=0.4	σ_μ=0.6
Power & Type I error	MANOVA	0.14	0.21	0.33	0.74	0.56	0.7
	aSPU.ind	0.04	0.14	0.33	0.04	0.16	0.36
	aSPU.ex	0.04	0.13	0.31	0.04	0.15	0.34
	TATES	0.05	0.08	0.2	0.06	0.38	0.59
	minP	0.05	0.1	0.26	0.05	0.43	0.64
	Fisher	0.05	0.13	0.36	0.05	0.39	0.57
	AFz	0.05	0.12	0.32	0.05	0.43	0.64
	AFp	0.05	0.12	0.34	0.05	0.42	0.63
Sensitivity	AFz	-	0.17	0.3	-	0.26	0.31
	AFp	-	0.18	0.33	-	0.3	0.42
Specificity	AFz	-	0.81	0.79	-	0.83	0.85
	AFp	-	0.81	0.78	-	0.8	0.77

Table S5: The average weight estimate for AFp and AFz methods for Simulation II. For example, for the AFp method, when $\sigma_\mu = 0.6$ and $N_1 = 100$, the average weight estimate for genes 1–1600 and 2md phenotype $\boldsymbol{Y_2}$ is 0.72 among 500 simulated data. Since the weight can be either 0 or 1, it means 72% of the weights for $\boldsymbol{Y_2}$ and genes 1–1600 are estimated to be 1 among 500 simulated datasets.

N	σ_μ	Method	Gene sets	$\boldsymbol{Y_1}$	$\boldsymbol{Y_2}$	$\boldsymbol{Y_3}$	$\boldsymbol{Y_4}$	$\boldsymbol{Y_5}$	$\boldsymbol{Y_6}$	$\boldsymbol{Y_7}$	$\boldsymbol{Y_8}$	$\boldsymbol{Y_9}$	$\boldsymbol{Y_{10}}$
$N_1 = 30$	$\sigma_\mu = 0.4$	AFp	$\boldsymbol{X_1 - X_{1600}}$	0.55	0.15	0.14	0.15	0.28	0.14	0.15	0.14	0.15	0.09
			$\boldsymbol{X_{1601} - X_{3200}}$	0.08	0.09	0.09	0.09	0.47	0.16	0.15	0.15	0.15	0.1
			$\boldsymbol{X_{3201} - X_{4800}}$	0.09	0.09	0.1	0.1	0.09	0.1	0.1	0.1	0.1	0.32
		AFz	$\boldsymbol{X_1 - X_{1600}}$	0.55	0.11	0.1	0.1	0.19	0.1	0.1	0.1	0.11	0.06
			$\boldsymbol{X_{1601} - X_{3200}}$	0.07	0.08	0.08	0.08	0.46	0.13	0.12	0.13	0.12	0.08
			$\boldsymbol{X_{3201} - X_{4800}}$	0.08	0.09	0.09	0.09	0.09	0.09	0.09	0.1	0.09	0.3
	$\sigma_\mu = 0.6$	AFp	$\boldsymbol{X_1 - X_{1600}}$	0.64	0.29	0.28	0.29	0.46	0.25	0.27	0.26	0.27	0.09
			$\boldsymbol{X_{1601} - X_{3200}}$	0.08	0.08	0.08	0.09	0.6	0.26	0.23	0.24	0.24	0.1
			$\boldsymbol{X_{3201} - X_{4800}}$	0.09	0.09	0.09	0.09	0.08	0.1	0.09	0.1	0.1	0.68
		AFz	$\boldsymbol{X_1 - X_{1600}}$	0.62	0.15	0.14	0.14	0.17	0.12	0.13	0.13	0.14	0.04
			$\boldsymbol{X_{1601} - X_{3200}}$	0.07	0.06	0.06	0.07	0.59	0.2	0.18	0.19	0.19	0.07
			$\boldsymbol{X_{3201} - X_{4800}}$	0.07	0.07	0.08	0.08	0.07	0.08	0.08	0.09	0.08	0.66
$N_1 = 100$	$\sigma_\mu = 0.4$	AFp	$\boldsymbol{X_1 - X_{1600}}$	0.66	0.34	0.33	0.32	0.65	0.34	0.33	0.33	0.34	0.1
			$\boldsymbol{X_{1601} - X_{3200}}$	0.07	0.1	0.1	0.1	0.67	0.3	0.29	0.29	0.28	0.09
			$\boldsymbol{X_{3201} - X_{4800}}$	0.09	0.1	0.09	0.1	0.09	0.1	0.09	0.09	0.09	0.75
		AFz	$\boldsymbol{X_1 - X_{1600}}$	0.64	0.15	0.15	0.14	0.18	0.15	0.14	0.15	0.15	0.04
			$\boldsymbol{X_{1601} - X_{3200}}$	0.05	0.05	0.05	0.05	0.66	0.17	0.16	0.17	0.16	0.05
			$\boldsymbol{X_{3201} - X_{4800}}$	0.07	0.08	0.07	0.08	0.08	0.07	0.07	0.07	0.07	0.73
	$\sigma_\mu = 0.6$	AFp	$\boldsymbol{X_1 - X_{1600}}$	0.85	0.72	0.71	0.71	0.84	0.7	0.67	0.69	0.68	0.11
			$\boldsymbol{X_{1601} - X_{3200}}$	0.08	0.1	0.1	0.1	0.83	0.64	0.63	0.64	0.63	0.09
			$\boldsymbol{X_{3201} - X_{4800}}$	0.08	0.1	0.08	0.09	0.08	0.09	0.08	0.08	0.08	0.99
		AFz	$\boldsymbol{X_1 - X_{1600}}$	0.77	0.28	0.27	0.27	0.25	0.26	0.25	0.25	0.25	0.03
			$\boldsymbol{X_{1601} - X_{3200}}$	0.03	0.03	0.03	0.03	0.79	0.28	0.28	0.29	0.28	0.03
			$\boldsymbol{X_{3201} - X_{4800}}$	0.02	0.03	0.02	0.02	0.02	0.02	0.02	0.02	0.02	0.99

Table S6: Results of Simulations IIIA and IIIB when $N_1 = 30$. For $\sigma_\mu = 0$, the value indicates Type I error control, and for $\sigma_\mu = 0.4$ and 0.6, the value indicates power.

Benchmark	Method	Simulation IIIA			Simulation IIIB		
		σ_μ=0	σ_μ=0.4	σ_μ=0.6	σ_μ=0	σ_μ=0.4	σ_μ=0.6
Power & Type I error	TATES	0.05	0.15	0.41	0.05	0.29	0.59
	minP	0.06	0.19	0.44	0.06	0.37	0.61
	Fisher	0.06	0.24	0.47	0.06	0.38	0.57
	AFz	0.06	0.22	0.51	0.06	0.4	0.67
	AFp	0.06	0.23	0.51	0.06	0.38	0.64
Sensitivity	AFz	-	0.25	0.44	-	0.26	0.39
	AFp	-	0.26	0.5	-	0.31	0.53
Specificity	AFz	-	0.81	0.8	-	0.79	0.79
	AFp	-	0.8	0.77	-	0.78	0.75

Table S7: The average weight estimate for AFp and AFz methods for Simulation III. For example, for the AFp method, when $\sigma_\mu = 0.6$ at $N_1 = 30$, the average weight estimate for genes 1–1600 and 2nd phenotype $\boldsymbol{Y_2}$ is 0.71 among 500 simulated data. Since the weight can be either 0 or 1, it means 73% of the weights for $\boldsymbol{Y_2}$ and genes 1–1600 are estimated to be 1 among 500 simulated datasets.

N	σ_μ	Method	Gene sets	$\boldsymbol{Y_1}$	$\boldsymbol{Y_2}$	$\boldsymbol{Y_3}$	$\boldsymbol{Y_4}$	$\boldsymbol{Y_5}$	$\boldsymbol{Y_6}$	$\boldsymbol{Y_7}$	$\boldsymbol{Y_8}$	$\boldsymbol{Y_9}$	$\boldsymbol{Y_{10}}$
$N_1 = 30$	$\sigma_\mu = 0.4$	AFp	$\boldsymbol{X_1} - \boldsymbol{X_{1600}}$	0.37	0.36	0.37	0.36	0.15	0.15	0.16	0.15	0.16	0.09
			$\boldsymbol{X_{1601}} - \boldsymbol{X_{3200}}$	0.13	0.12	0.12	0.11	0.15	0.17	0.16	0.17	0.17	0.09
			$\boldsymbol{X_{3201}} - \boldsymbol{X_{4800}}$	0.12	0.12	0.11	0.11	0.09	0.09	0.09	0.09	0.09	0.36
		AFz	$\boldsymbol{X_1} - \boldsymbol{X_{1600}}$	0.33	0.33	0.34	0.33	0.14	0.14	0.16	0.14	0.15	0.09
			$\boldsymbol{X_{1601}} - \boldsymbol{X_{3200}}$	0.11	0.1	0.1	0.1	0.14	0.16	0.15	0.16	0.16	0.09
			$\boldsymbol{X_{3201}} - \boldsymbol{X_{4800}}$	0.1	0.11	0.1	0.1	0.08	0.09	0.09	0.09	0.08	0.34
	$\sigma_\mu = 0.6$	AFp	$\boldsymbol{X_1} - \boldsymbol{X_{1600}}$	0.7	0.71	0.72	0.72	0.27	0.27	0.29	0.27	0.29	0.1
			$\boldsymbol{X_{1601}} - \boldsymbol{X_{3200}}$	0.14	0.13	0.12	0.12	0.26	0.3	0.28	0.29	0.3	0.09
			$\boldsymbol{X_{3201}} - \boldsymbol{X_{4800}}$	0.12	0.13	0.12	0.11	0.09	0.09	0.08	0.09	0.08	0.72
		AFz	$\boldsymbol{X_1} - \boldsymbol{X_{1600}}$	0.58	0.6	0.6	0.6	0.2	0.21	0.23	0.22	0.23	0.08
			$\boldsymbol{X_{1601}} - \boldsymbol{X_{3200}}$	0.11	0.1	0.1	0.1	0.24	0.29	0.27	0.28	0.29	0.08
			$\boldsymbol{X_{3201}} - \boldsymbol{X_{4800}}$	0.09	0.1	0.09	0.09	0.07	0.07	0.07	0.08	0.07	0.71
$N_1 = 100$	$\sigma_\mu = 0.4$	AFp	$\boldsymbol{X_1} - \boldsymbol{X_{1600}}$	0.79	0.78	0.79	0.79	0.31	0.32	0.32	0.31	0.32	0.09
			$\boldsymbol{X_{1601}} - \boldsymbol{X_{3200}}$	0.12	0.12	0.11	0.13	0.31	0.3	0.32	0.3	0.32	0.1
			$\boldsymbol{X_{3201}} - \boldsymbol{X_{4800}}$	0.11	0.11	0.11	0.12	0.09	0.1	0.1	0.1	0.1	0.76
		AFz	$\boldsymbol{X_1} - \boldsymbol{X_{1600}}$	0.66	0.65	0.65	0.66	0.21	0.21	0.22	0.21	0.22	0.06
			$\boldsymbol{X_{1601}} - \boldsymbol{X_{3200}}$	0.1	0.1	0.09	0.11	0.28	0.28	0.3	0.28	0.29	0.09
			$\boldsymbol{X_{3201}} - \boldsymbol{X_{4800}}$	0.08	0.08	0.08	0.09	0.07	0.08	0.07	0.08	0.07	0.74
	$\sigma_\mu = 0.6$	AFp	$\boldsymbol{X_1} - \boldsymbol{X_{1600}}$	1	1	1	1	0.64	0.68	0.7	0.68	0.7	0.09
			$\boldsymbol{X_{1601}} - \boldsymbol{X_{3200}}$	0.11	0.1	0.1	0.1	0.6	0.63	0.65	0.63	0.64	0.1
			$\boldsymbol{X_{3201}} - \boldsymbol{X_{4800}}$	0.09	0.08	0.08	0.1	0.08	0.1	0.09	0.09	0.08	0.99
		AFz	$\boldsymbol{X_1} - \boldsymbol{X_{1600}}$	0.87	0.87	0.87	0.87	0.25	0.3	0.31	0.3	0.32	0.02
			$\boldsymbol{X_{1601}} - \boldsymbol{X_{3200}}$	0.05	0.05	0.05	0.05	0.5	0.54	0.57	0.54	0.56	0.07
			$\boldsymbol{X_{3201}} - \boldsymbol{X_{4800}}$	0.02	0.01	0.01	0.02	0.02	0.02	0.02	0.02	0.02	0.99

Table S8: The pathway enrichment analysis of each module by GO, KEGG, and Reactome pathway databases for the brain aging dataset with samples from human prefrontal cortex (Brodmann's area 11).

pathway	pvalue
module 1 (innate immune system)	
Reactome complement cascade	1.33e-04
KEGG complement and coagulation cascades	2.39e-04
Reactome initial triggering of complement	2.50e-04
GO:CC endoplasmic reticulum	5.82e-03
GO:MF nucleoside-triphosphatase activity	9.87e-03
GO:BP histone modification	9.89e-03
GO:MF extracellular matrix structural constituent	1.06e-02
Reactome innate immune system	1.21e-02
GO:MF protein serine/threonine kinase activity	1.25e-02
GO:MF hydrolase activity, acting on acid anhydrides	1.39e-02
module 2 (immune signaling)	
KEGG lysine degradation	3.60e-03
Reactome interferon alpha/beta signaling	5.45e-03
KEGG regulation of autophagy	8.80e-03
Reactome regulation of IFNA signaling	1.01e-02
Reactome TRAF6 mediated IRF7 activation	1.29e-02
Reactome transcriptional regulation of white adipocyte differentiation	1.60e-02
GO:MF oxidoreductase activity, acting on paired donors, with incorporation or reduction of molecular oxygen	1.77e-02
Reactome interferon signaling	2.37e-02
GO:MF cytokine receptor binding	2.50e-02
GO:MF enzyme inhibitor activity	2.58e-02
Module 3 (cell signalling)	
GO:MF rhodopsin-like receptor activity	1.61e-04
GO:MF ras gtpase activator activity	4.54e-04
Reactome glycosaminoglycan metabolism	5.31e-04
GO:MF G-protein-coupled receptor activity	5.59e-04
Reactome class A/1 (rhodopsin-like receptors)	1.08e-03
Reactome GPCR–ligand binding	1.11e-03
GO:MF small gtpase regulator activity	1.56e-03
GO:BP G-protein-coupled receptor signaling pathway	1.76e-03
GO:BP cell surface receptor signaling pathway	2.55e-03
Reactome G-alpha (q) signalling events	2.58e-03

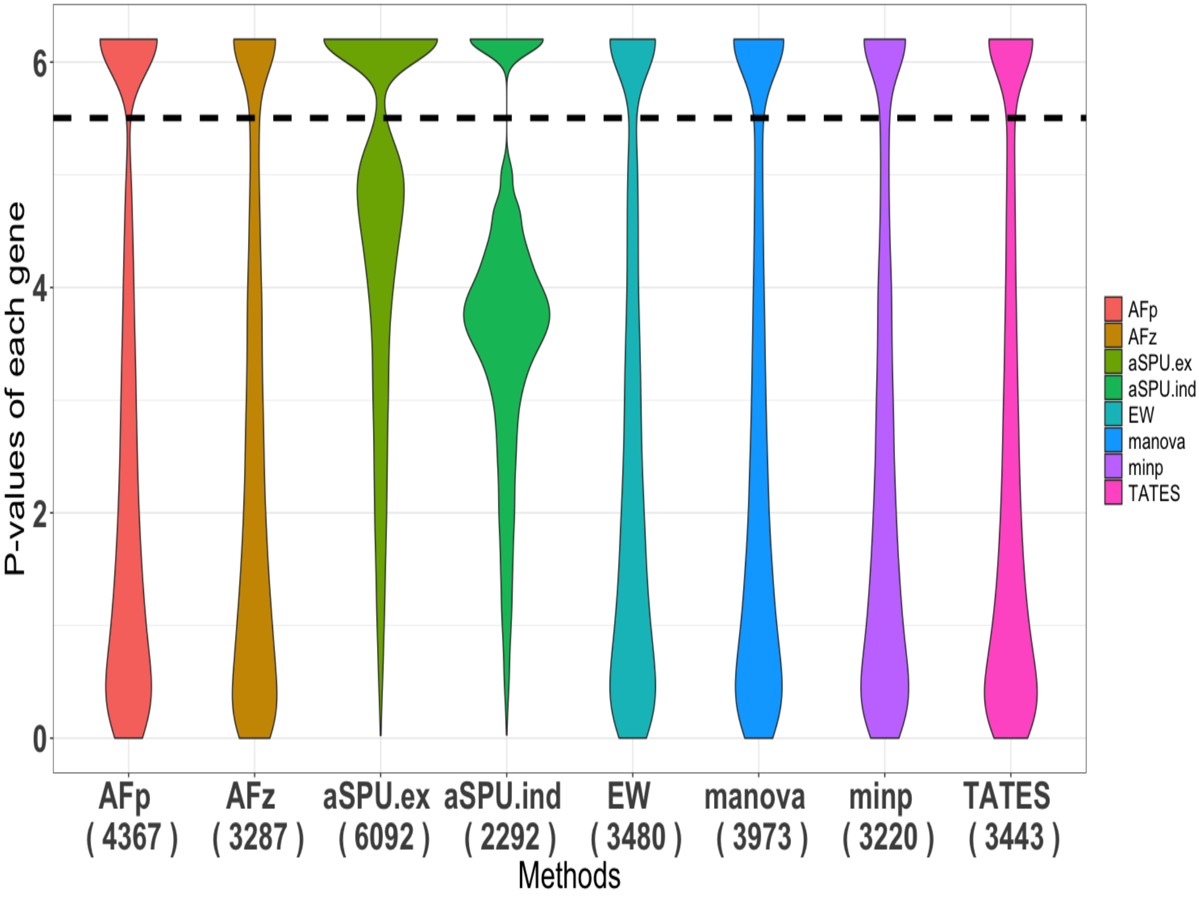

Figure S1: $-\log_{10}(p\text{-value})$for all the methods for the lung disease dataset. The number in the parenthesis is the number of significant genes determined by Bonferroni correction with cutoff 0.05.

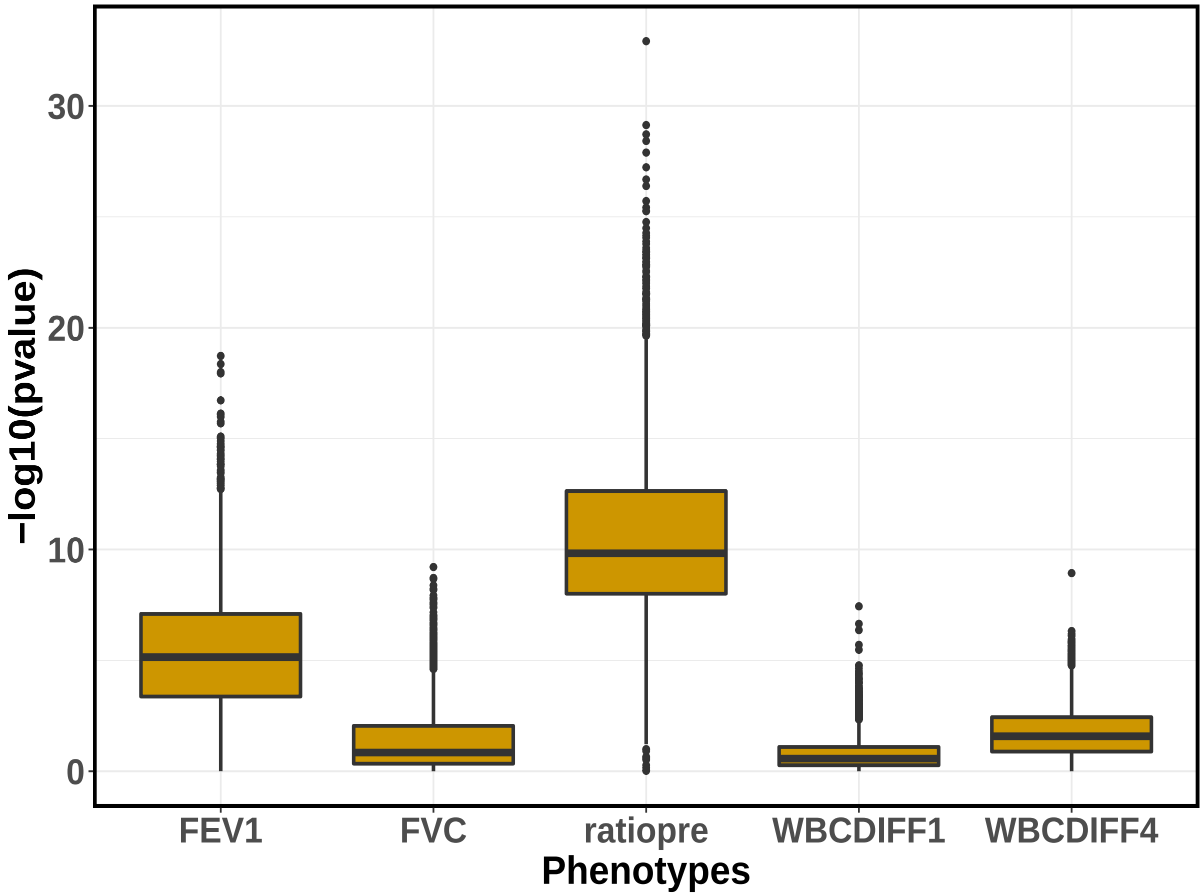

Figure S2: Boxplot of $-\log_{10}(p\text{-value})$ of significant genes for each phenotype detected by both AFp and AFz methods for the lung disease dataset.

Figure S3: The heatmap shows the $-\log_{10}(p_{jk}) \times \text{sign}(\theta_{jk})$ for jth gene and kth phenotype among the genes identified in tight clustering for the lung disease dataset. We truncate $-\log_{10}(p_{jk}) \times \text{sign}(\theta_{jk})$ to $[-10, 10]$ for better visualization. Green (-10) means a gene is negatively associated with the phenotype, and red (10) means a gene is positively associated with the phenotype.

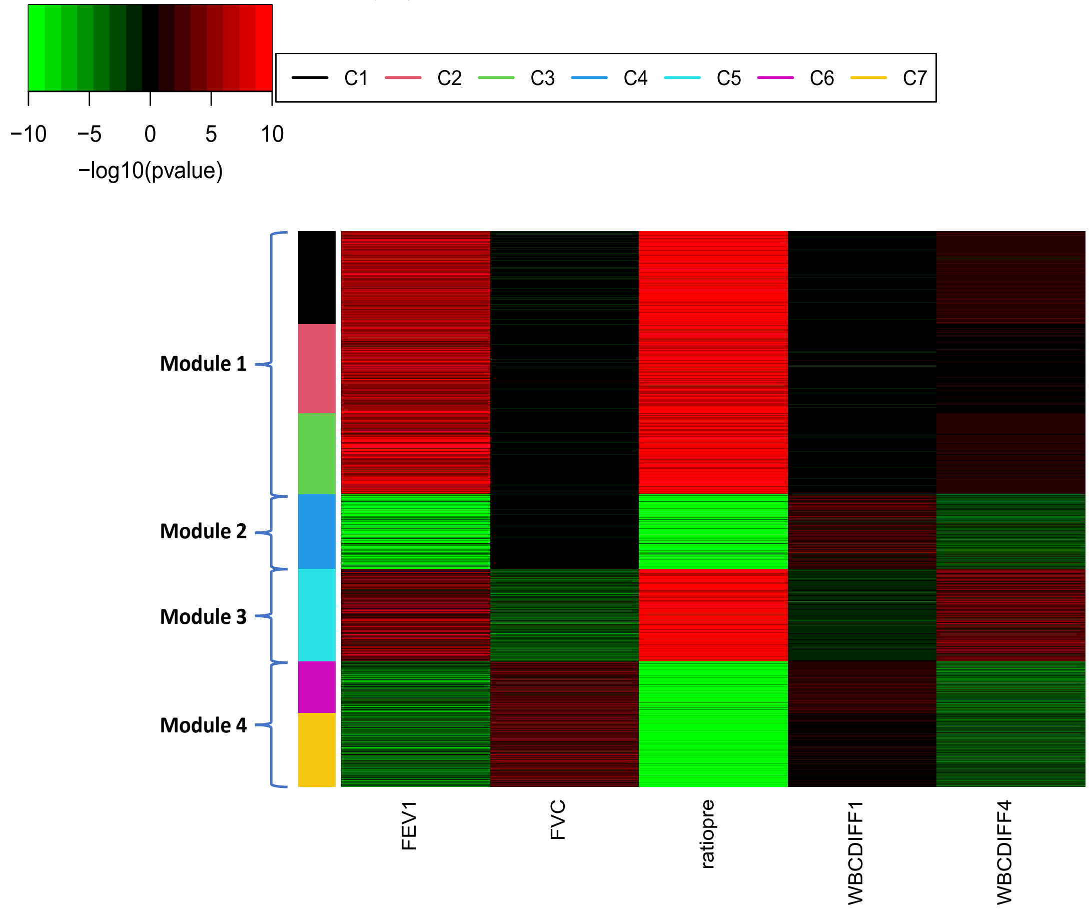

Figure S4: Box plots of Spearman's correlations between phenotypes and gene expressions of genes in the seven clusters (C1–C7) for the lung disease dataset.

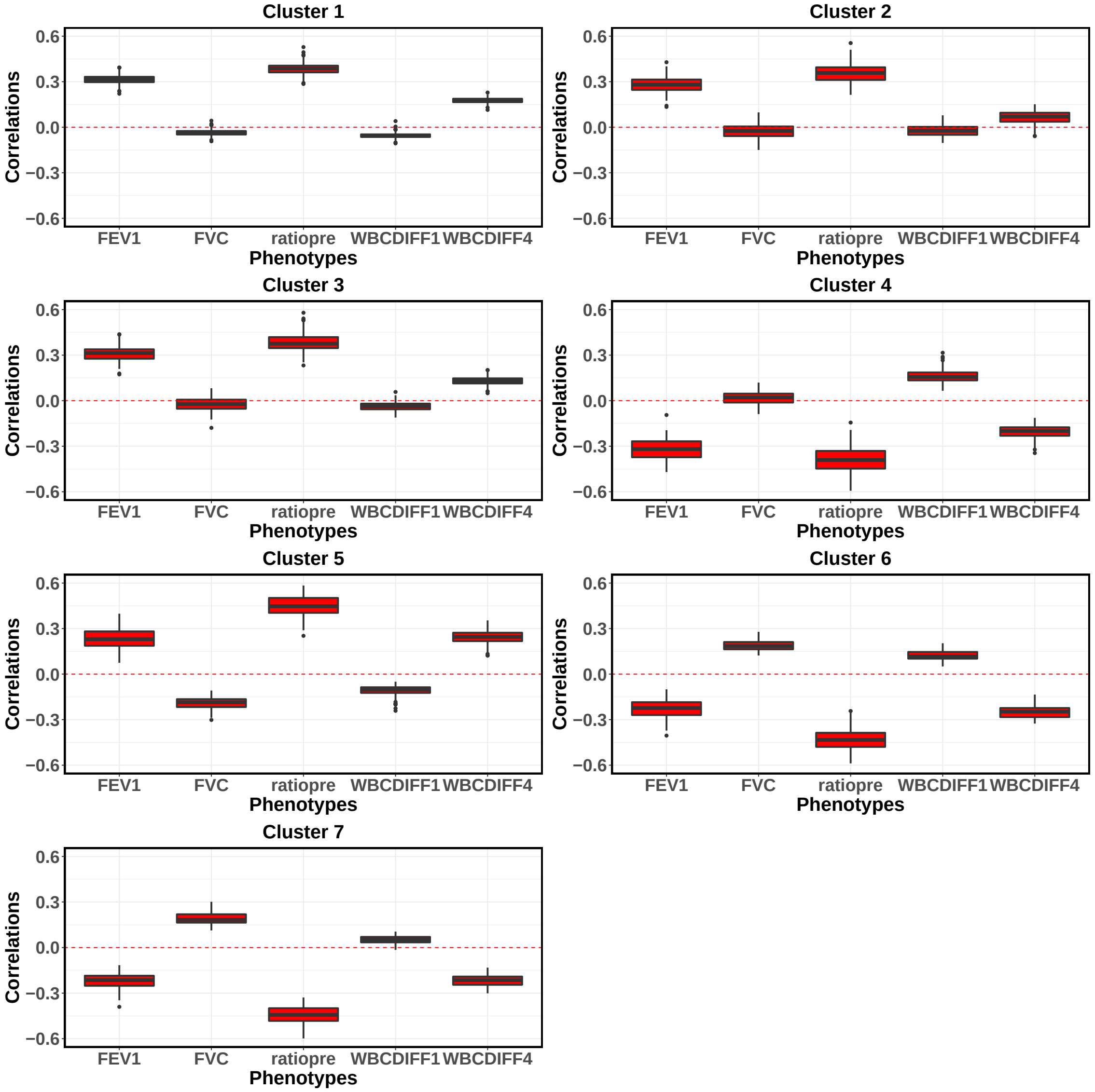

Figure S5: Heatmap of the variability indices for detected genes for the lung disease dataset.

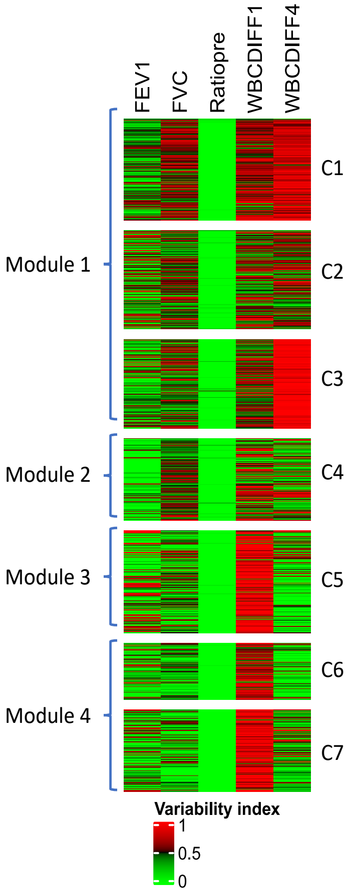

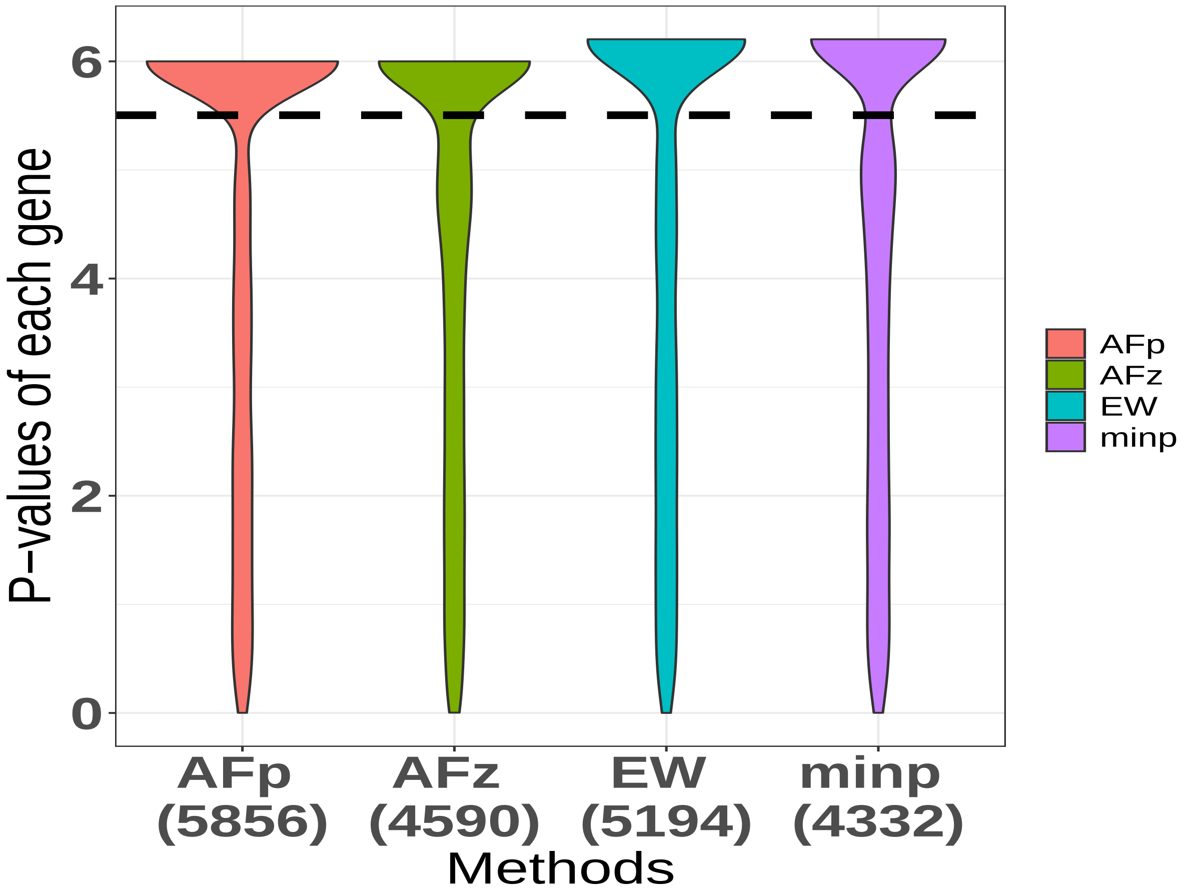

Figure S6: $-\log_{10}(p\text{-value})$for the four methods that can be applied to the breast cancer dataset (METABRIC) with a mixture of phenotypes of different data types. The number in the parenthesis is the number of significant genes determined by Bonferroni correction with cutoff 0.05.

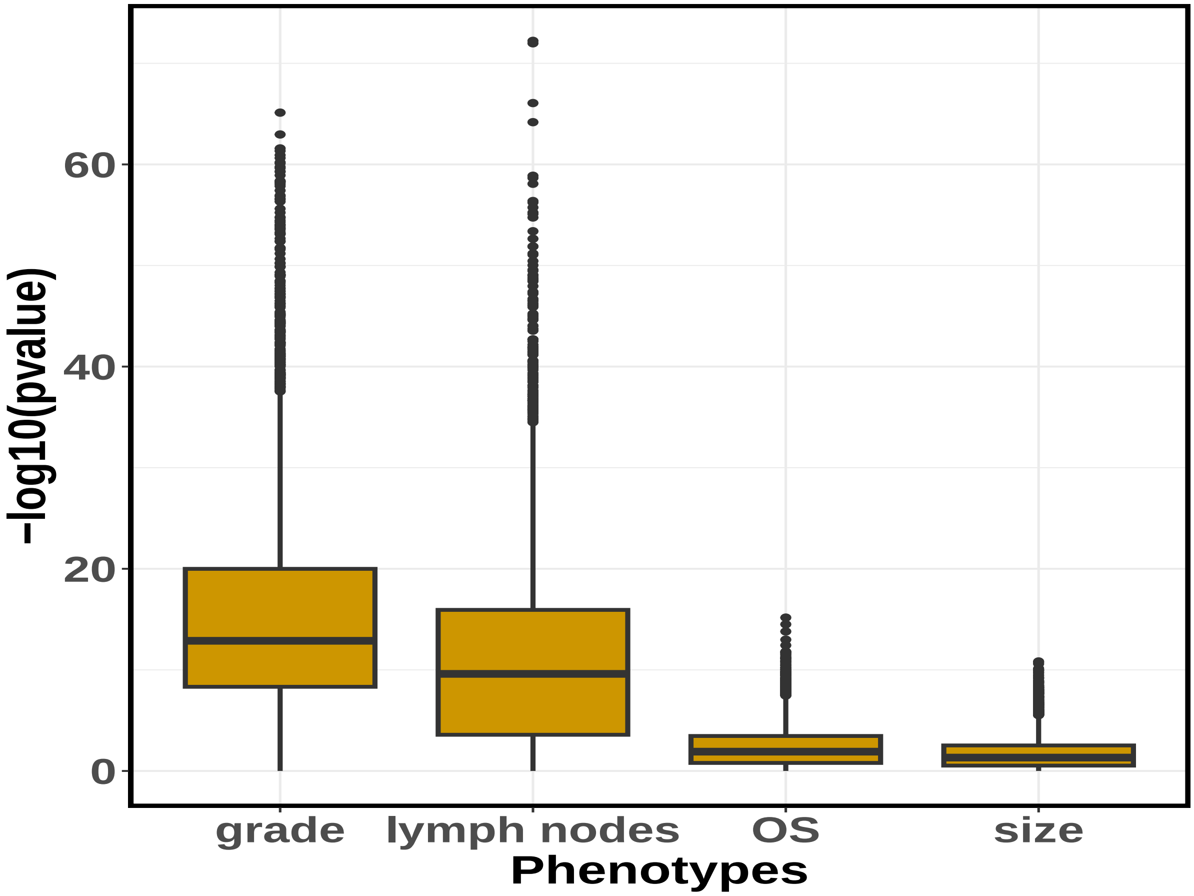

Figure S7: Boxplot of $-\log_{10}(p\text{-value})$ of significant genes for each phenotype detected by both AFp and AFz methods for the breast cancer dataset (METABRIC).

Figure S8: The heatmap shows the $-\log_{10}(p_{jk}) \times \text{sign}(\theta_{jk})$ for the jth gene and kth phenotype among the genes identified in tight clustering for the breast cancer dataset. We truncate $-\log_{10}(p_{jk}) \times \text{sign}(\theta_{jk})$ to $[-10, 10]$ for better visualization. Green (-10) means a gene is negatively associated with the phenotype, and red (10) means a gene is positively associated with the phenotype.

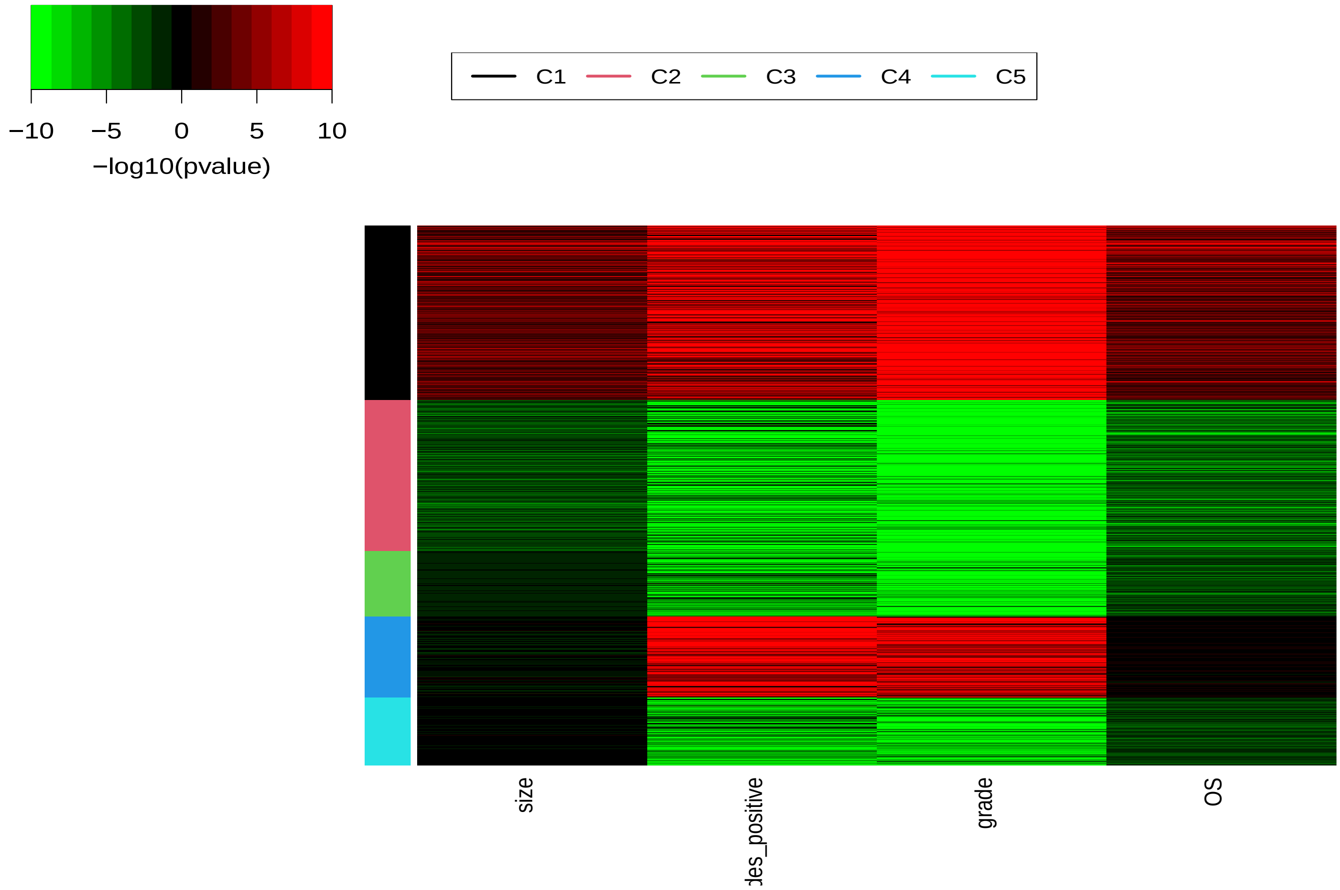

Figure S9: Box plots of Spearman's correlations between phenotypes and gene expressions of genes in the five clusters (C1–C5) for the breast cancer dataset (METABRIC).

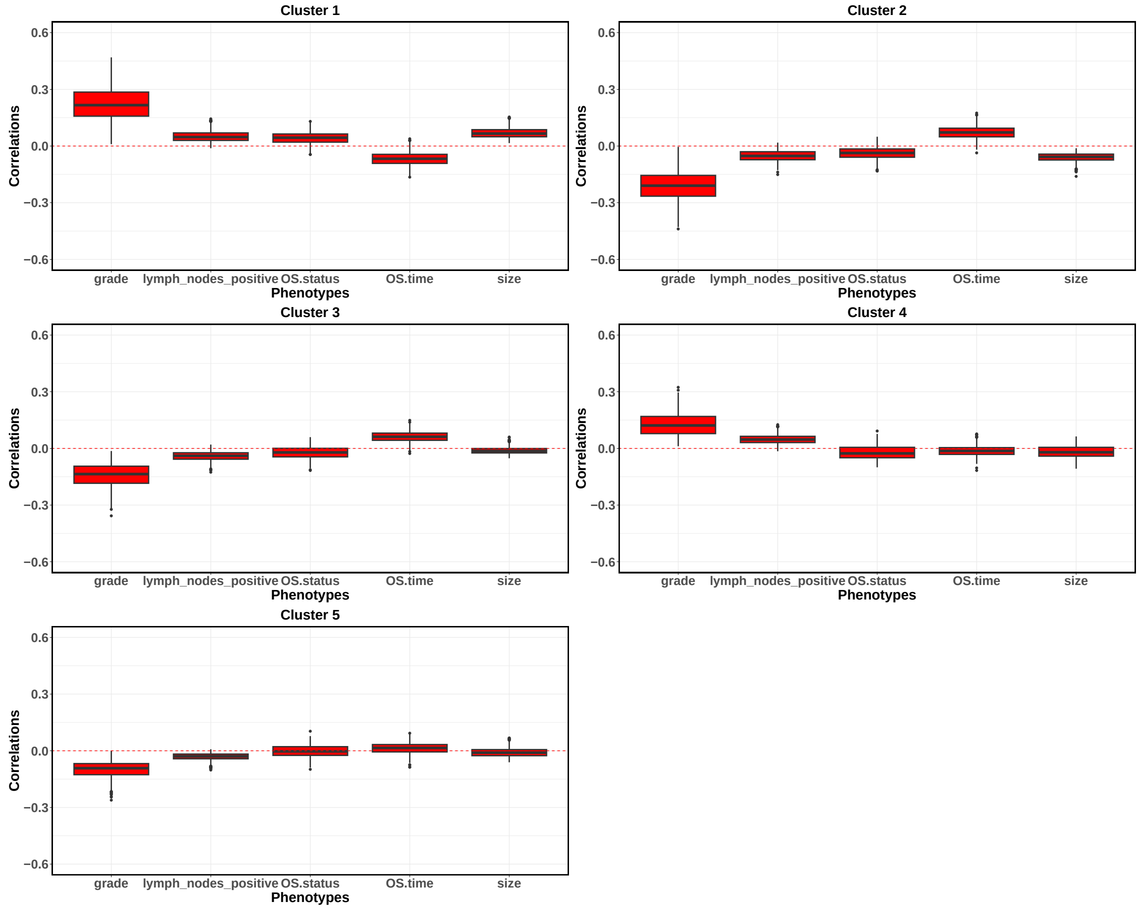

Figure S10: Heatmap of the variability indices for detected genes for the breast cancer dataset (METABRIC).

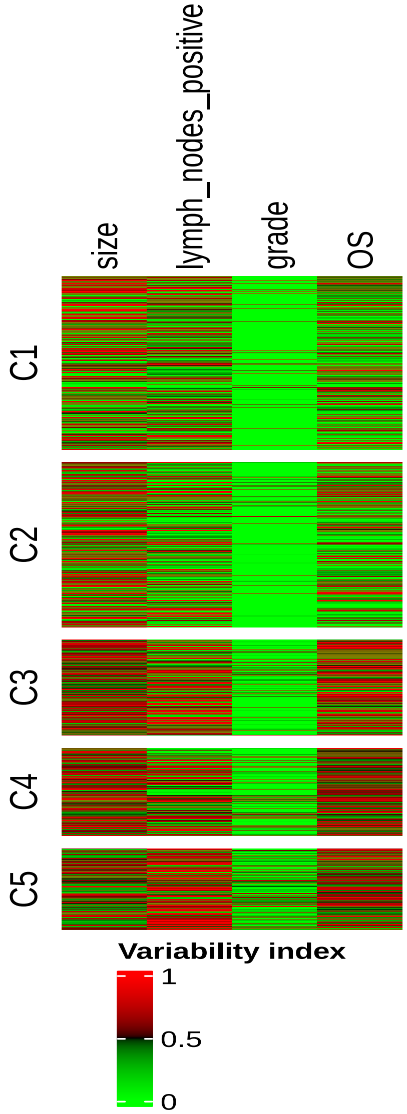

Figure S11: (a) The heatmap of the co-membership matrix of three clusters identified in the brain aging dataset. Red color means two genes are close. The number in the parentheses indicates the sample size of each cluster. (b) The weight estimation of each gene (blue represent 1, black represents 0, and yellow represents -1).

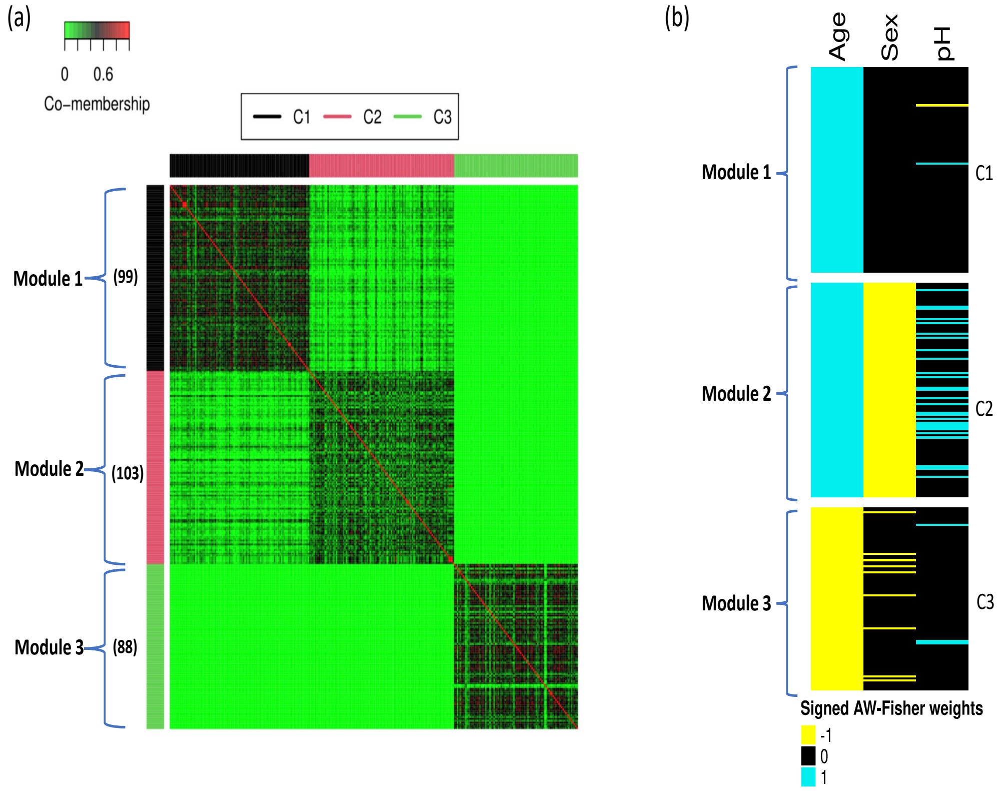

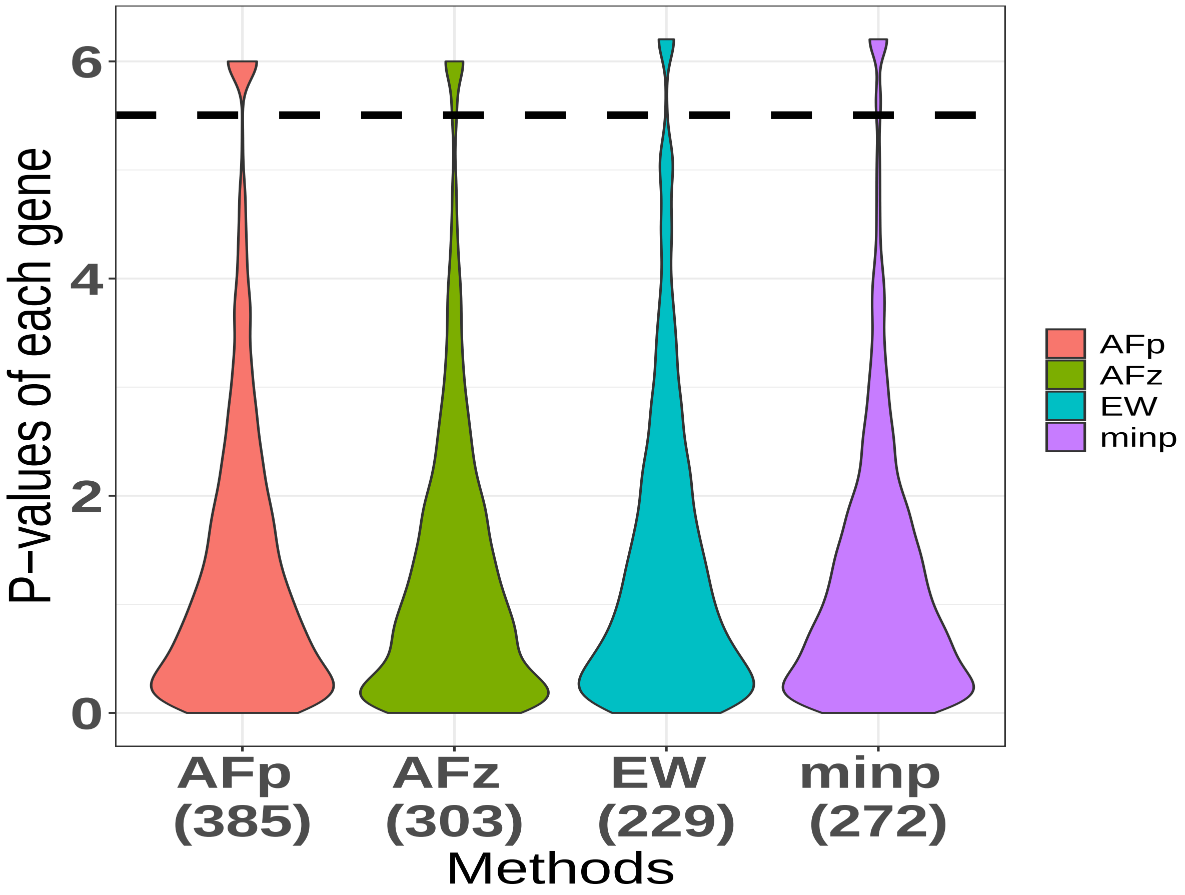

Figure S12: $-\log_{10}(p\text{-value})$ for the four methods that can be applied to the brain aging dataset with a mixture of phenotypes of different data types. The number in the parenthesis is the number of significant genes determined by Benjamini–Hochberg correction with cutoff 0.05.

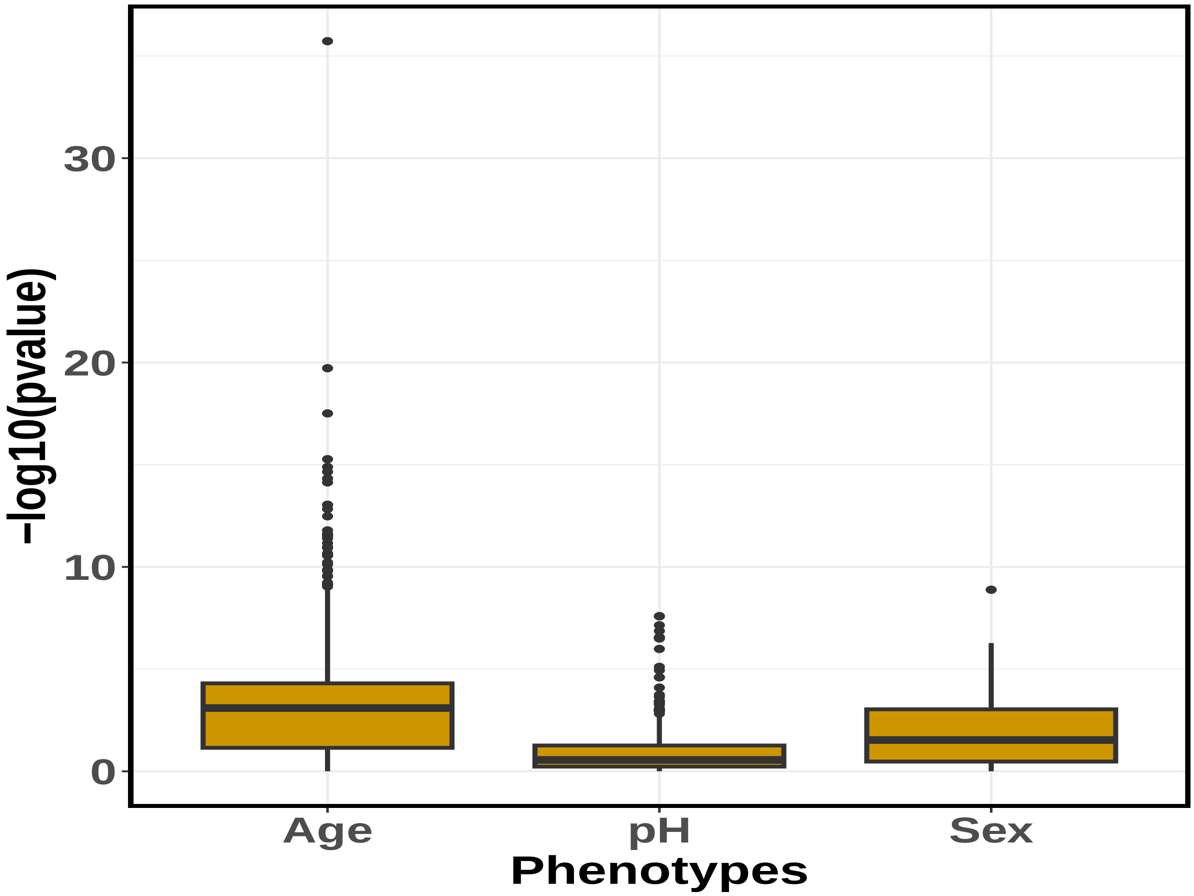

Figure S13: Boxplot of $-\log_{10}(p\text{-value})$ of significant genes for each phenotype detected by both AFp and AFz methods for the brain aging dataset.

Figure S14: The heatmap shows the $-\log_{10}(p_{jk}) \times \text{sign}(\theta_{jk})$ for jth gene and kth phenotype among the genes identified in tight clustering for the brain aging dataset. We truncate $-\log_{10}(p_{jk}) \times \text{sign}(\theta_{jk})$ to $[-10, 10]$ for better visualization. Green (-10) means a gene is negatively associated with the phenotype, and red (10) means a gene is positively associated with the phenotype.

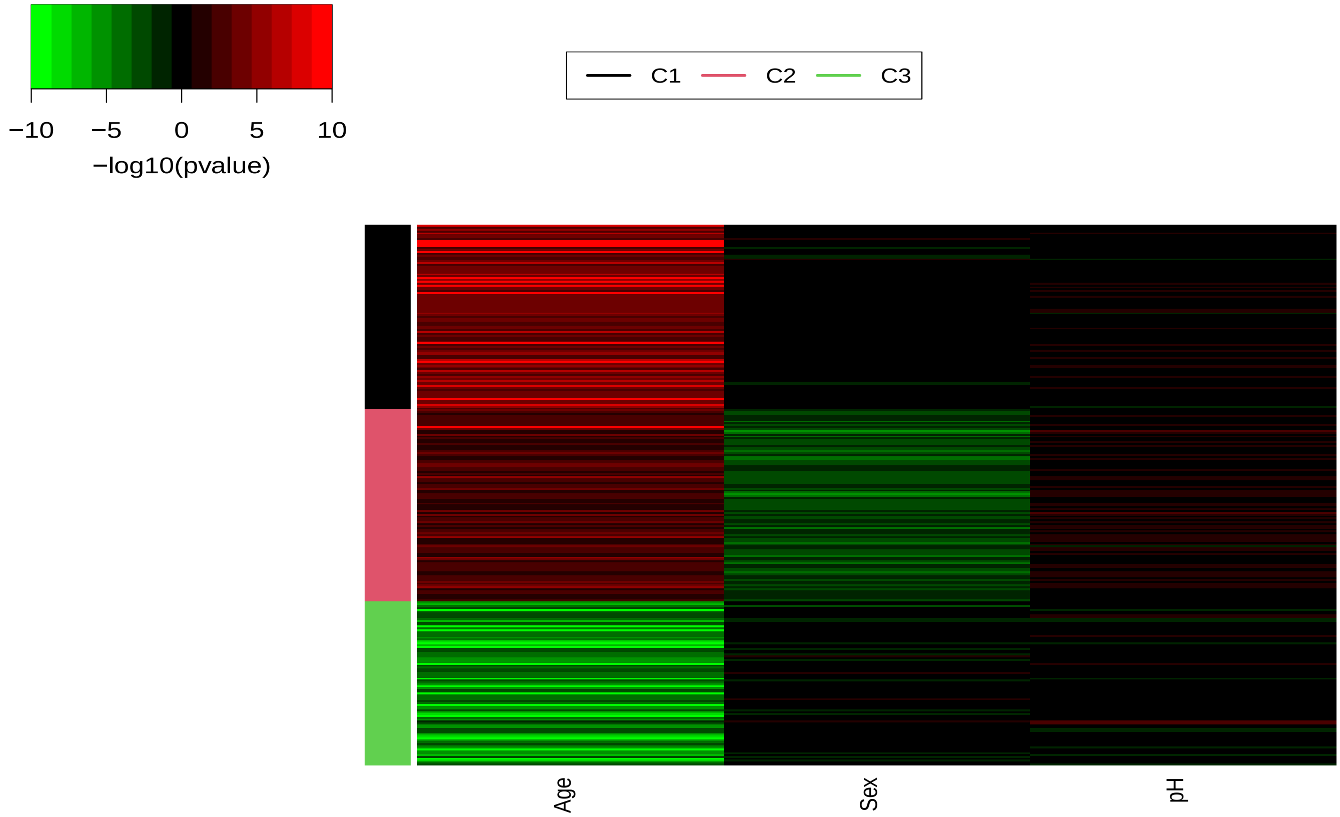

Figure S15: Box plots of Spearman's correlations between phenotypes and gene expressions of genes in the three clusters (C1-C3) for the brain aging dataset.

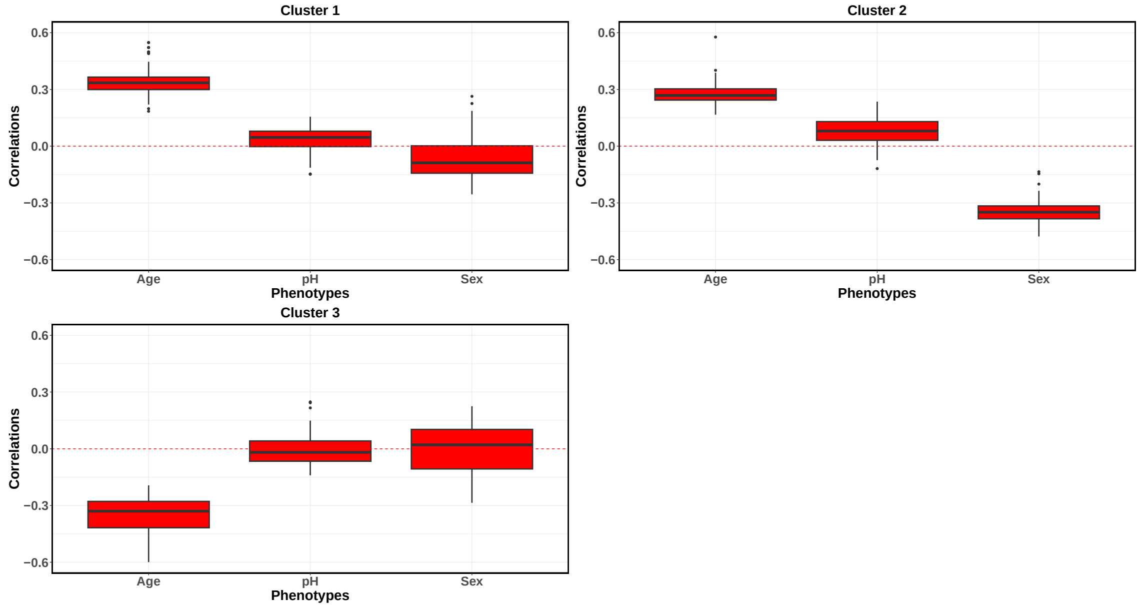

Figure S16: Heatmap of the variability indices for detected genes for the brain aging dataset.

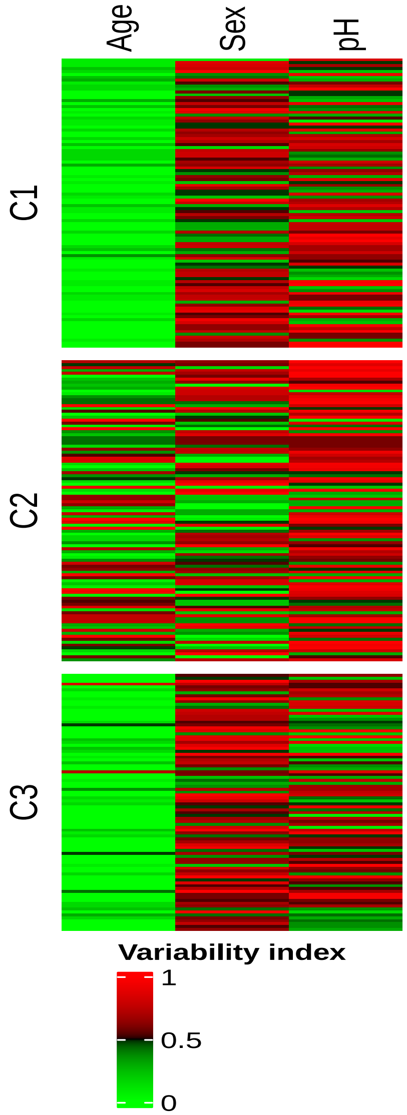

Supplement: Supplementary file 1 [file genes-14-00798-s001.zip › genes-2191088-supplementary.pdf]
